# Welcome Address

**Published:** 1872-05

**Authors:** V. H. Taliaferro

**Affiliations:** Columbus, Georgia


### WELCOME ADDRESS

*To the Georgia Medical Association, April 10th, 1872, at Columbus, Georgia.*

BY V. H. TALIAFERRO, M.D., COLUMBUS, GEORGIA.

*Gentlemen of the Georgia Medical Association,*—It is my privilege and pleasure, in behalf of the physicians of Columbus, to extend to you their cordial welcome, and to assure you that they are proud and honored to have you in their midst. Representing, as you do, every section of Georgia, and constituting, in your organized capacity, one of the most learned and scientific bodies which has ever convened within her borders, we could not be otherwise than honored by your presence.

In consideration of your high objects and aims, embodying everything within the purview of your deliberations that pertains to human suffering, your Association is fraught with a peculiar interest to every individual, however rich or poor, however proud or humble. You are here, that by your united wisdom, medical science may be advanced in the collection of facts and the establishment of great truths. It is a matter, then, of no surprise that this entire community have looked forward to your coming with an anxious and a profound interest.

Your deliberations are so intimately blended with the dearest interests of communities, and so interwoven with benevolence and charity, that every man, woman and child in the country becomes a participant in the results of your labors, whether for good or for evil. Let us trust, then, that every effort will be used to make this meeting of your Association the most harmonious and profitable that has ever assembled.

Associations such as yours constitute the greatest pillars of the medical profession, and to these must we look for effective barriers to stay the tide of epidemics which sweep their alarming and horrid devastations over communities and countries. In the last decade of years, wonderful results have been achieved by hygiene and quarantine, in holding at bay, upon the waters of our coast, the kindling fires of cholera and yellow fever, and turning them back to their native haunts. May we not confidently look to the day when these destructive pests of the human family shall be as greatly modified in their virulence and fatality as has been small-pox by vaccination. The prediction of the immortal Jenner—that through the agency of vaccination, small-pox should eventually be eradicated from the face of the globe—may never be fully verified; yet we now see, that in place of its being the most dreaded of all diseases, carrying off its hundreds of thousands yearly, the monster has become as a child, no longer to be dreaded by physicians or communities. The real nature of the poisons which germinate exotic diseases, though still cloaked in obscurity, are nevertheless brought within the compass of successful quarantine.

Our profession, gentlemen, through your organized labors and by your individual labors, has of late years made wonderful advances in all the departments of medicine—especially in physiology, pathology, and therapeutics. Within the past thirty years, the practical application of anæsthesia, by ether and chloroform, has shed immortal lustre upon the medical profession, and made renowned the names of the discoverers. Those who have tasted the blessings of chloroform under the most trying ordeals of human flesh, and the physicians who witness the melting away of pain into blissful forgetfulness, can fully appreciate this heavenly boon to man.

Again: within the past few years, we have chloral to fill a blank so long and painfully felt by the profession.

And still again: electro-therapeutics, though now in its cradle, promises to become a giant in its maturity.

And yet again: within so few years past, gynæcology has taken rank as an important and distinct branch of medicine.

Time and opportunity will not, of course, permit us here to even enumerate the more recent and important advances made of late in medicine. But, gentlemen, though you have accomplished so very much by your associated labors—though you have been rewarded by the lights of science in many dark places—there are yet questions of vast magnitude before you, veiled in impenetrable darkness. Tuberculosis, cancer, scrofula, are still draped in mystery, in defiance of all the resources of science, and the diligent and laborious investigations of past ages. Let us trust that the time is not distant when some great master in medicine shall proclaim to us the real nature of these diseases.

But recently, the news comes to us from Vienna of a discovery by Dr. Lostorfer, in connection with the most lamentable blight of the human family, and which, if true, must result eventually in inestimable blessings, and perchance may constitute an important epoch in a branch of medicine which, for all time past, has been clouded by vague speculations and theories. This gentleman has, by the examination of the blood of the affected individuals, brought within the field of his microscope a peculiar diagnostic fungi. These experiments were verified by Prof. Hebra, and a number of distinguished doctors and professors in medicine.

It will be remembered that, several years since, Prof. Saulsbury, of Cincinnati, announced, in the *American Journal of Medical Sciences*, the discovery of what he termed *cryptogamic spores* in the blood of the diseased persons, and which he claimed as invariably diagnostic.

These discoveries, if sustained by facts, will, in all probability, be found to differ only in name, and if so, will give us but another evidence of the transcendency of American genius; and it, may be, will constitute the first link in the chain by which is to be worked out the great problems of tuberculosis, scrofula, and cancer.

Among other important questions, gentlemen, which for years have been agitating your organizations, is that of *reform in medical education.* Learned essays have been read you from your national body, medical teachers' conventions have assembled year after year, and yet nothing definite has been accomplished to remedy the shortcomings and evils complained of. Many of us become impatient and disheartened at this slow progress, and ready to believe that the American Medical Association has undertaken a work to which it is incompetent. My own humble opinion is, that the radical changes which are proposed, and which are necessary, will be perfected so soon as the profession and the country are rife for them, and demand them.

Popular education, which at this day is attracting universal and profound interest, will in a few years give us enlightened and educated communities, and will stamp its requirements upon the profession and the people. We know too well how often it is that the merest charlatan in medicine, the most ignorant licensed pretender, competes successfully in patronage with the man of learning. Ignorance can have no understanding or appreciation of wisdom, but fosters ignorance; hence it is, that among an uneducated people, the medical man will be most liberally patronized whose mental caliber is in unison with their own. While we would confess that it is of the first moment that the medical schools of the country should have an established system of rigid preliminary educational requirements—lengthened and increased collegiate terms, together with an increased number of teachers—we are convinced that the educational status and requirements of the country will exercise a powerful and controlling influence in this much-desired and greatly-needed reformation.

Again, gentlemen, let me assure you of your cordial welcome, and of our earnest desire to make your stay in our city agreeable and pleasant to you as a body and as individuals.

